# Gene-Diet Interactions in Childhood Obesity

**DOI:** 10.2174/138920211795677903

**Published:** 2011-05

**Authors:** William S Garver

**Affiliations:** Department of Biochemistry and Molecular Biology, The University of New Mexico Health Sciences Center, Albuquerque, New Mexico, USA

**Keywords:** Adolescent, childhood, gene-diet, high-fat, nutrition, obesity, overweight.

## Abstract

Childhood overweight and obesity have reached epidemic proportions worldwide, and the increase in weight-associated co-morbidities including premature type 2 diabetes mellitus (T2DM) and atherosclerotic cardiovascular disease will soon become major healthcare and economic problems. A number of studies now indicate that the childhood obesity epidemic which has emerged during the past 30 years is a complex multi-factorial disease resulting from interaction of susceptibility genes with an obesogenic environment. This review will focus on gene-diet interactions suspected of having a prominent role in promoting childhood obesity. In particular, the specific genes that will be presented (FTO, MC4R, and NPC1) have recently been associated with childhood obesity through a genome-wide association study (GWAS) and were shown to interact with nutritional components to increase weight gain. Although a fourth gene (APOA2) has not yet been associated with childhood obesity, this review will also present information on what now represents the best characterized gene-diet interaction in promoting weight gain.

## CHILDHOOD OVERWEIGHT AND OBESITY

The Centers for Disease Control and Prevention (CDC) defines childhood overweight as a body mass index (BMI) equal to and greater than the age and gender specific 85th percentile (BMI ≥ 85th percentile), where BMI is calculated as weight in kilograms divided by height in meters squared (BMI = kg/m^2^) [1]. This definition of childhood overweight includes the category of childhood obesity defined as a BMI equal to and greater than the age and gender specific 95th percentile (BMI ≥ 95th percentile). The most recent National Health and Nutrition Examination Survey (NHANES) indicates that childhood (2-11 years of age) overweight and obesity in the United States has approximately doubled during the past three decades, while adolescent (12-19 years of age) overweight and obesity has more than tripled during the same period [2]. This study also indicates that 9.5% of infants/toddlers (birth-2 years of age) and 16.9% of children/adolescents were at or above the 95th percentile of BMI, therefore categorized as being obese. On a global scale, a recent World Health Organization (WHO) nutritional survey obtained from 43 million children living in 144 different countries indicates that preschool childhood overweight and obesity increased from 4.2% in 1990 to 6.7% in 2010, with prevalence expected to reach approximately 9.1% by 2020 [3]. Childhood and adolescent overweight and obesity (referred to hereafter as childhood obesity) has reached epidemic proportions both nationally and internationally; consequently, increased weight-associated co-morbidities, including premature type 2 diabetes mellitus (T2DM) and atherosclerotic cardiovascular disease, will soon represent major healthcare and economic problems [4]. 

For decades the primary causes of childhood obesity remained unknown, due in part to uncertainty about whether weight gain was caused by psychological or biological (genetic) factors [5]. For instance, in the early to middle parts of the twentieth century, studies suggested that psychological factors were primarily responsible for specific behavior (overconsumption of food) contributing to childhood obesity [6]. However, other studies performed using twins fed different diets and/or raised independently, in addition to the eventual cloning of a specific gene (leptin gene) associated with early-onset weight gain, suggested that genetic factors were also responsible for childhood obesity [7, 8]. But since the childhood obesity epidemic is a new phenomenon that has emerged only during the past 30 years, it is unlikely that genetic variation alone has significantly contributed to this healthcare problem. It is important to realize that the timing of this epidemic parallels increased availability of calorie-dense foods and a more sedentary lifestyle, in what is now commonly referred to as an “obesogenic environment”. Therefore, although genetic changes are not responsible for the increase in childhood obesity, the condition is probably the result of genetic susceptibility manifested in an obesogenic environment, most likely through complex and undefined gene-diet and gene-physical activity interactions. 

## THRIFTY GENES AND INTERACTION WITH AN OBESOGENIC ENVIRONMENT

A presentation of gene-environment interactions should involve the concept of “thrifty genes”, which was first introduced by the American geneticist James V. Neel in relation to an enhanced predisposition to T2DM among certain groups of people [9]. However, since publication of his original article, the vast majority of citations have been in reference to obesity. In brief, it was hypothesized that certain indigenous people with hunter-gatherer lifestyles experienced repeated periods of feast and famine, which through adaptation resulted in the natural selection of thrifty genes. These thrifty genes permitted efficient conversion of food calories into fat when food supplies were plentiful (feast portion of the cycle) and that fat was later used as an energy source when food was limited (famine portion of the cycle). However, the selective advantage of these thrifty genes soon became a disadvantage when these people were exposed to a chronic obesogenic environment characterized by a high-fat diet and sedentary lifestyle; in essence the continual and excessive storage of fat prepared these people for a famine that never arrived. As a result, the existence of thrifty genes among certain groups of people, the most studied and documented of which include the Pima Indians of Arizona, may be responsible for excessive weight gain and associated T2DM [10, 11]. Although the concept of thrifty genes remains valid, there has recently been much debate concerning potential fundamental flaws involving the original hypothesis [12, 13]. The controversial points include whether hunter-gatherers actually experienced severe enough famines for natural section of thrifty genes, and if severe enough famines happened to exist whether natural selection could have been mediated through other means such as fertility, instead of viability [13, 14]. As a result of these debates, additional and more comprehensive hypotheses have emerged. These hypotheses include a network of five non-mutually exclusive genotypes (thrifty, hyperphagic, sedentary, low lipid oxidation, and adipogenesis), in addition to the possible involvement of genome plasticity proposing that genes may interact with components in the current obesogenic environment to increase weight gain and obesity [15, 16].

Finally, since the human population is genetically diverse and heterogenous with over 7.7 million single nucleotide polymorphisms (SNPs), identifying and investigating potential gene-diet interactions in relation to various human diseases including obesity is further complicated by gene-gene interactions. The many different and simultaneous gene-gene interactions therefore contribute to what is commonly referred to as a multi-factorial or polygenic disease. As a result, to circumvent this inherent difficulty in identifying and investigating gene-diet interactions related to weight gain and obesity, researchers have necessarily used genetically identical mouse models that possess only one specific gene variant of interest. 

## GENE-DIET INTERACTIONS CONTRIBUTING TO CHILDHOOD OBESITY

Although a number of genes are associated with either weight gain or obesity, few have definitively been shown to interact with diet and contribute to early-onset or childhood obesity [17]. One particular GWAS recently identified the first genes associated with early-onset (before 6 years of age) and extreme childhood and morbid adult obesity (BMI ≥ 40 kg/m^2^) [18]. In brief, the study used nearly 1,400 obese Europeans compared to a similar number of age-matched normal weight controls for the first step or discovery stage, in addition to 2,100 obese and 2,400 normal weight individuals for the second step or confirmatory stage, and then 9,700 individuals from the general population for the third step or re-confirmatory stage. The five genes found to be associated with childhood obesity included the fat mass and obesity associated (*FTO*) gene, the melanocortin-4 receptor (*MC4R*) gene, the Niemann-Pick C1 (*NPC1*) gene, the musculoaponeurotic fibrosarcoma (*MAF*) gene, and the phosphotriesterase-related (*PTER*) gene. Recent studies indicate that three of these genes (*FTO*, *MC4R*, and *NPC1*) interact with a high-fat diet to promote early-onset or childhood obesity. Therefore, a more comprehensive review of these specific genes will be presented in this article. Moreover, although the apolipoprotein A-2 (*APOA2*) gene has not been found to be associated with childhood obesity in a GWAS, this review will also present information on what now represents the best characterized gene-diet interaction in promoting increased weight gain. It is important to emphasize that due to the complexity of elucidating gene-diet interactions in a heterogeneous human population, studies performed using mouse models will be emphasized. 

## THE FAT MASS AND OBESITY ASSOCIATED GENE

**Fig. (1):**
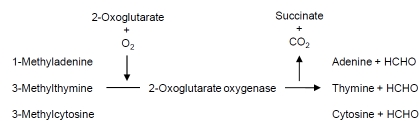
The biochemical reaction catalyzed by 2-oxoglutarate oxygenase encoded by the *FTO* gene is responsible for demethylating certain nucleic acid bases (1-methyladenine, 3-methylthymine, and 3-methylcytosine) when associated with single-stranded RNA or DNA. Studies indicate that *FTO* gene variants interact with the diet by increasing preference for a specific nutrient (fat) and decreasing satiety to promote weight gain.

The fat mass and obesity associated (*FTO*) gene represents the first gene identified that contributes to common forms of human obesity [19]. The *FTO* gene, located on the long arm of human chromosome 16 within cytogenetic band q12.2, encodes a protein classified within the non-heme dioxygenase (Fe(II) and 2-oxoglutarate-dependent dioxygenase) superfamily of proteins due to extensive conservation of amino acid sequence [20, 21]. The encoded FTO protein is localized to the nucleus where it catalyzes demethylation of certain nucleic acid bases (1-methyladenine, 3-methylthymine, and 3-methylcytosine) associated with single-stranded RNA or DNA (Fig. **1**). The *FTO* gene is ubiquitously expressed in both mouse and human tissues, with the highest amounts of messenger RNA found in the arcuate hypothalamus and regulated by fasting/feeding cycles. This provided the first evidence that the FTO protein possessed an important regulatory role in maintaining energy balance [20, 22]. Although the molecular basis for how the FTO protein maintains energy balance remains undefined, two recent studies suggest that *FTO* gene variants are associated with promoting increased weight gain. In the first of these studies, overweight individuals (BMI ≥ 25 kg/m^2^) who were either heterozygous or homozygous for a common *FTO* gene variant (rs9939609) had significantly decreased cerecortical beta-activity compared to lean individuals (BMI ≤ 25 kg/m^2^) without the *FTO* gene variant [23]. This result suggested that individuals with the *FTO* gene variant had brain insulin resistance, consistent with a previous study demonstrating that proper insulin-induced brain signaling was necessary for satiety and appetite control in maintaining energy balance [24]. In the second study, subcutaneous adipose tissue biopsies obtained from a cohort of 306 healthy women (BMI = 18-53 kg/m^2^) indicated that individuals heterozygous for the common *FTO* gene variant had increased *in vitro* adipocyte glycerol release (22%) and *in vivo* plasma glycerol levels (30%), suggesting that the FTO protein had an undefined role in regulating the hydrolysis of triacyglycerol within adipocytes [25].

The corresponding *Fto* gene in mice was first identified as a result of generating a transgenic mouse model that coincidently caused deletion of a genomic region containing the *Fto* gene [26]. Several years later, a GWAS designed to identify genes associated with T2DM in both adults and children detected the *FTO* gene, which after adjustment for BMI revealed that the *FTO* gene was actually associated with BMI and not T2DM [19]. Another GWAS published the same year with 442 lean and 487 extremely obese German children/adolescents indicated that the *FTO* gene had a strong association with extreme early-onset or childhood obesity [27]. Moreover, another study performed using 234 full-term healthy newborns (112 boys and 122 girls) also indicated an association between *FTO* gene variants, abdominal fat mass, and body weight at only two weeks of age [28]. The most recent GWAS has confirmed that the *FTO* gene, in addition to two other obesity susceptibility genes (*MC4R* and *NPC1*, reviewed below) are associated with childhood and adult obesity within a European population [18]. However, not all individuals possessing *FTO* gene variants are overweight or obese, suggesting that possible interactions with other genetic and/or environmental factors could be necessary for promoting weight gain and obesity [29].

Recent studies provide valuable insight into the molecular basis for how *FTO* gene variations promote weight gain. In brief, *FTO* gene variants in both children and adults are associated with increased energy consumption and decreased satiety [30, 31]. Consistent with these results, studies performed using *Fto* deficient mice (*Fto*^-/-^) indicated a relative increase in energy consumption [32]. These results provided the first evidence indicating that *FTO* gene variants may interact with environmental factors, such as the diet, to promote weight gain. With respect to a potential *FTO* gene-diet interaction in promoting increased BMI, a cross-sectional study of 4,839 adults demonstrated a significant interaction between the common *FTO* gene variant and dietary fat in relation to increased BMI after adjustment for total energy consumption [33]. Moreover, another study performed using 289 children and adolescents between 6-19 years of age with the common *FTO* gene variant indicated that although total energy consumption did not differ significantly from controls, individuals with the *FTO* gene variant consumed a greater percentage of calories derived from fat [34]. These results indicate that individuals who possess *FTO* gene variants have an increased preference for calorie-dense foods enriched with fat and decreased satiety, factors responsible for promoting weigh gain and childhood obesity.

## THE MELANOCORTIN 4-RECEPTOR GENE

**Fig. (2):**
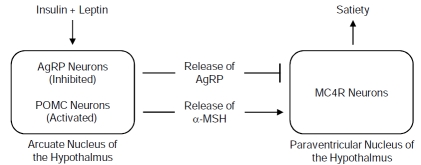
The insulin and leptin-melanocortin signaling pathway is responsible for maintaining energy and metabolic balance by controlling appetite. The melanocortin-4 receptor (MC4R) encoded by the *MC4R* gene binds to peptide hormones (α-MSH or AgRP) secreted from either α-MSH or AgRP expressing neurons, respectively, present in the arcuate nucleus of the hypothalamus. Studies indicate that *MC4R* gene variants interact with the diet by increasing preference for a specific nutrient (fat) and decreasing satiety to promote increased weight gain. AgRP, agouti-related peptide; POMC, proopiomelanocortin; α-MSH, α-melanocyte stimulating hormone.

The melanocortin 4-receptor (*MC4R*) gene is responsible for the most common forms of childhood and adult obesity. The *MC4R* gene, located on the long arm of human chromosome 18 within cytogenetic band q21.32, encodes a large and complex plasma membrane protein belonging to the family of seven transmembrane G protein-coupled receptors that activate the adenylate cyclase-dependent pathway responsible for cAMP signaling [35, 36]. The *MC4R* gene is primarily expressed in the paraventricular nucleus of the hypothalamus, where an endogenous agonist (alpha-melanocyte stimulating hormone or α-MSH) and antagonist (agouti-related protein or AgRP) compete for binding to the MC4R protein [37]. Activation of the MC4R protein by binding α-MSH results in decreased feeding and increased energy expenditure through thermogenesis, while deactivation of the MC4R protein by binding AgRP inhibits this process and therefore decreases satiety and energy expenditure [38]. Secretion of insulin and leptin, both found to be directly associated with the amount of adipose tissue, has an indirect role in maintaining energy balance through the MC4R pathway by stimulating synthesis of proopiomelanocortin (POMC) from POMC neurons, which is then processed to α-MSH capable of being bound by the MC4R protein (Fig. **2**) [39]. As might be expected, genetic variation resulting in loss of function for the *MC4R*, *LEP*, and *POMC* genes contributes to weight gain and obesity.

The first definitive study demonstrating that the *MC4R* gene has a role in promoting obesity was performed by targeted disruption of this gene (*Mc4r*) within mice; this explained excessive weight gain in what was commonly referred to as the agouti mouse model [40, 41]. In this study, the *Mc4r* heterozygous (*Mc4r^+/-^*) mice were characterized with intermediate weight gain when compared to *Mc4r* normal (*Mc4r^+/+^*) and Mc4r homozygous (*Mc4r^-/-^*) mice, indicating codominant inheritance. Soon after, two different frameshift mutations were identified in a severely obese child (10 years of age, BMI = 34 kg/m^2^) and a non-related obese middle aged woman (35 years of age, BMI = 30 kg/m^2^) known to have developed obesity during infancy [42, 43]. Consistent with studies performed using *Mc4r* mice, the first large study performed using patients with *MC4R* mutations indicated a codominant mode of inheritance [44]. A number of later studies then identified three types of *MC4R* gene variants, which included more than a hundred loss-of-function mutations responsible for promoting obesity, two gain-of-function mutations protecting from obesity, and a frequent intergenic polymorphism associated with a modest increase in risk for obesity [42, 45]. Together, these *MC4R* gene variants have not only been associated with promoting and protecting from obesity, but also with traits related to the intake and preference for foods. However, similar to the *FTO* gene, not all individuals possessing *MC4R* gene variants are overweight or obese, again suggesting that possible interactions with other genetic and/or environmental factors could be necessary for promoting weight gain and obesity.

In comparison to *Mc4r^+/+^* mice fed a moderately high-fat diet, the *Mc4r^-/-^* mice were found to have hyperphagia, in addition to a partial defect in energy expenditure characterized by decreased diet-induced activity and thermogenesis [46, 47]. Consistent with these results, another study demonstrated that compared to *Mc4r^+/+^* mice, the *Mc4r^-/-^* mice had hyperphagia when fed a high-fat diet, but not when fed a low-fat diet. This finding provided the first evidence for a gene-diet interaction in relation to weight gain in these mice [48]. An extension of these studies demonstrated that *Mc4r^-/-^* mice fed a high-fat diet developed severe hepatic steatosis and insulin resistance, characterized in part by impaired fasting glucose and insulin tolerance, suggesting that these mice were also susceptible to features associated with T2DM [49]. Furthermore, recent studies have indicated that individuals possessing *MC4R* gene variants tend not only to consume increased amounts of food, but also prefer specific nutrients (total and saturated fat) [50]. A genome-wide linkage study performed to identify chromosomal regions contributing to increased dietary consumption and decreased physical activity among 1,030 Hispanic children has provided strong evidence that *MC4R* gene variants have a role in regulating body weight through both energy consumption and expenditure [51, 52]. Although the molecular basis remains undefined and penetrance varies considerably among individuals, these results are consistent with individuals that possess *MC4R* gene variants having an increased preference for calorie-dense foods enriched with fat and decreased satiety responsible for promoting weight gain and childhood obesity.

## THE NIEMANN-PICK C1 GENE

**Fig. (3):**
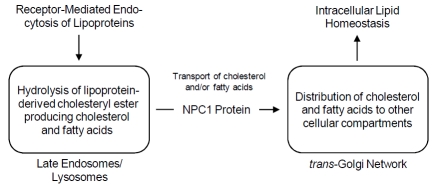
The NPC1 protein encoded by the *NPC1* gene regulates the transport of lipids (cholesterol and fatty acids) from late endosomes/lysosomes to other cellular compartments to maintain intracellular, tissue, and whole body lipid homeostasis. Although human-based studies have not yet been performed investigating the molecular basis for the *NPC1* gene-diet interaction, studies performed using an NPC1 mouse model indicates that decreased *Npc1* gene dosage or *Npc1* haploinsufficiency resulting from a null mutation interacts with a high-fat diet to promote weight gain.

The Niemann-Pick C1 (*NPC1*) gene has been primarily investigated in relation to an autosomal-recessive lipid-storage disorder characterized by neonatal jaundice, hepatosplenomegaly, and progressive neurodegeneration resulting in death during the second decade [53, 54]. The *NPC1* gene, located on the long arm of human chromosome 18 within cytogenetic band q11.2 near the centromere, encodes a large and complex multi-spanning transmembrane protein with extensive structural homology with members of the resistance-nodulation-division (RND) family of prokaryotic permeases [55, 56]. Considering the various structural motifs, the NPC1 protein contains 13 membrane-spanning helices and 3 large luminal domains, among which an N-terminal domain (NTD) and sterol-sensing domain (SSD) independently bind cholesterol [57, 58]. The NPC1 protein is associated with a unique late endosomal compartment that transiently interacts with low-density lipoprotein (LDL)-derived cholesterol enriched late endosomes/lysosomes; it facilitates the transport of cholesterol and possibly other lipids (fatty acids) to various cellular compartments, including the *trans*-Golgi network, plasma membrane, and endoplasmic reticulum [59, 60]. Although the exact function of the NPC1 protein remains undefined, studies have demonstrated that expression of the *NPC1* gene is regulated through the sterol regulatory element-binding protein (SREBP) pathway, consistent with the NPC1 protein having a central role in maintaining cellular, tissue, and whole body lipid homeostasis (Fig. **3**) [61, 62].

In retrospect, a number of early studies performed using both *NPC1* human fibroblasts and the BALB/cJ *NPC1* mouse model revealed information concerning the potential involvement of the NPC1 protein in maintaining energy balance. For instance, *NPC1* heterozygous (*NPC1^+/-^*) human fibroblasts have increased expression of caveolin-1, which serves as a protein marker for obesity and T2DM [63, 64]. These results were confirmed and extended using *Npc1* heterozygous (*Npc1^+/-^*) mice, which compared to *Npc1* normal (*Npc1^+/+^*) and *Npc1* homozyous (*Npc1^-/-^*) mice, had livers with an increased expression of caveolin-1 and concentration of triacylglycerol, which is a prominent metabolic feature of obesity [65-67]. Together, these results were consistent with increased expression of caveolin-1 having a central role in the transport of fatty acids (not cholesterol) and storage of triacylglycerol within lipid storage bodies involved in maintaining energy balance [68, 69]. 

A recent GWAS revealed that the *NPC1* gene was associated with early-onset and morbid adult obesity in European populations, but researchers did not know whether the *NPC1* gene non-synonymous SNPs (rs1805081 and rs1805082) increased or decreased corresponding NPC1 protein function. At that time our group was performing a candidate-gene based mouse study using the *NPC1* mouse model, which possesses a retroposon insertion resulting in a null mutation characterized by a severely truncated and non-functional NPC1 protein [70, 71]. The results from this study demonstrated that when compared to *Npc1*^+/+^ mice, the *Npc1*^+/-^ mice were characterized with significant weight gain when fed a high-fat diet, but not when fed a low-fat diet, consistent with a gene-diet interaction [72]. However, it must be emphasized, that similar to human NPC1 disease, the *Npc1*^-/-^ mice suffer from neurological degeneration, weight loss, and die before adulthood. To confirm the presence of an *Npc1* gene-diet interaction in relation to weight gain in the *Npc1^+/-^* mice, comprehensive statistical analyses of the growth data was performed using both fitted growth trajectories and estimated marginal means of body weight. The results demonstrated that only *Npc1*^+/-^ mice fed a high-fat diet (not a low-fat diet) were susceptible to weight gain, thereby providing the first evidence of a gene-diet interaction in relation to weight gain [73].

To further investigate the *Npc1* gene-diet interaction in relation to weight gain and associated metabolic features, BALB/cJ *Npc1*^+/-^ mice were interbred with wild-type C57BL/6J mice; the latter mouse strain commonly used to study aspects of diet-induced obesity and T2DM [74, 75]. This breeding produced a hybrid (BALB/cJ-C57BL/6J) *NPC1 *mouse model, for which the *Npc1^+/-^* mice were characterized with weight gain, abdominal adiposity, adipocyte hypertrophy, hepatic steatosis, plasma dyslipidemia, and impaired glucose tolerance in the absence of hyperphagia [76]. Results from this second study were similar to the initial study using the BALB/cJ *NPC1* mouse model, since an *Npc1* gene-diet interaction was found to promote both weight gain and metabolic features associated with insulin resistance. Although further studies are required, these results are consistent with decreased *Npc1* gene dosage or *Npc1* haploinsufficiency in mice interacting with a high-fat diet to either increase lipogenesis and/or storage of fat, or decrease energy expenditure (thermogensis and/or activity) to promote weight gain. 

## THE APOLIPOPROTEIN A-2 GENE

The apolipoprotein A-2 (*APOA2*) gene is a member of the apolipoprotein multigene (*APOA1*, *APOA2*, and *APOA4*) family that shares a common genomic structure and possesses a range of diverse and complex physiological functions that remain undefined. The *APOA2* gene, located on the long arm of human chromosome 1 within cytogenetic band q23.3, encodes a protein that is primarily associated with high density lipoprotein (HDL) in plasma [77]. Although a number of early studies suggested that the APOA2 protein had a functional role in regulating the transport of cholesterol from peripheral tissues back to the liver for excretion, subsequent studies were unable to confirm these results [78, 79]. To date, the physiological function for the APOA2 protein is instead believed to have a central role in regulating fatty acid and triacylglycerol metabolism by modulating lipoprotein lipase-mediated hydrolysis of triacylglycerol [80, 81]. 

**Fig. (4):**
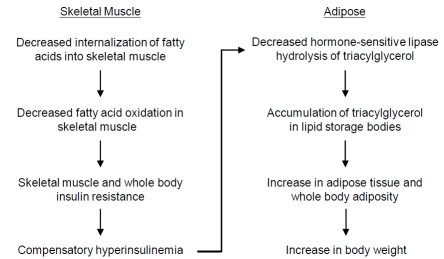
The APOA2 protein encoded by the *APOA2* gene has an undefined role in regulating fatty acid and triacylglyerol metabolism in at least two tissues (skeletal muscle and adipose) to maintain energy balance. Although the molecular basis remains undefined, studies indicate that the *APOA2* gene variants interact with saturated fats to promote weight gain in diverse populations of adult individuals.

Since it was determined that the APOA2 protein had a role in regulating fatty acid and triacylglyerol metabolism, subsequent studies were performed investigating the APOA2 protein in relation to adiposity and weight gain using both mouse models and humans. Studies using *Apoa2* transgenic mice expressing increased amounts of APOA2 protein showed that in addition to promoting increased liver lipogenesis, the APOA2 protein inhibited hydrolysis of triacylglycerol stored within adipose tissues (epididymal and retroperitoneal), thereby accounting for weight gain in these mice [82]. A subsequent study demonstrated that metabolic dysregulation present in the *Apoa2* transgenic mice resulted from reduced skeletal muscle fatty acid oxidation, skeletal muscle insulin resistance, and whole body compensatory hyperinsulinemia [83]. Researchers proposed that hyperinsulinemia increased both hepatic lipogenesis and secretion of VLDL resulting in hypertriacylglycerolemia and reduced hydrolysis of triacylglycerol in adipose tissues (Fig.**4**). Together, these results were consistent with an earlier study performed using *Apoa2* knock-out (Apoa2^-/-^) mice characterized with insulin hypersensitivity, decreased plasma triacyglycerol, and normal body weight [84]. Moreover, cross-breeding experiments using different mouse strains to identify genetic loci indicated that the *Apoa2* gene was associated with increased mouse adiposity and body weight [85, 86]. With respect to initial studies performed using humans, an *APOA2* SNP (rs5082) located within the *APOA2* promoter region resulting in decreased amounts of APOA2 protein was associated with decreased waist circumference in men, but increased waist circumference in women, both in the absence of significant differences in either body weight or BMI [87, 88]. A later study investigating the same *APOA2* SNP reported that individuals who were *APOA2* heterozygous (*APOA2^+/-^*) and *APOA2* homozygous (*APOA2^-/-^*) had a lower postprandial response (decreased postprandial hypertriacylglycerolemia) compared to individuals who were *APOA2* normal (*APOA2^+/+^*) when consuming a high saturated-fat diet with no significant difference noted for body weights between the group of individuals [89]. Therefore, although *Apoa2* mouse models had clearly demonstrated that increased expression of the APOA2 protein was associated with increased adiposity and weight gain, the physiological role of APOA2 protein in humans remained controversial due to seemingly contradictory results. 

More recent studies of the *APOA2* gene clarified the role of the APOA2 protein in regulating adiposity and body weight by demonstrating that the *APOA2* gene interacts with a high-fat diet to promote weight gain and obesity. The first of these studies, the Genetics of Lipid Lowering Drugs and Diet Network (GOLDN) study, used 1,078 individuals (514 men and 564 women) [90]. The results indicated that *APOA2^-/-^* individuals consumed increased amounts of food composed of fat and protein, in addition to having an increased body weight, BMI, and waist circumference compared to either *APOA2^+/+^* or *APOA2*^+/-^ individuals. Together these results suggested that the APOA2 protein served to regulate satiety and appetite control. A second study was then performed using 3,462 individuals obtained from 3 independent populations (Framingham Offspring Study with 1454 whites, GOLDN study with 1078 whites, and Boston-Puerto Rican Centers on Population Health and Health Disparities study with 903 Hispanics). The study was specifically designed to search for interactions occurring between the *APOA2* SNP (rs5082) and consumption (≥ 22 g per day) of saturated fat [91]. The results indicated a significant interaction between *APOA2^-/-^* individuals and consumption of saturated fat in relation to an increase in BMI for all 3 populations. These individuals had a 6.2% greater increase in BMI when consuming a high-saturated fat diet, but not when consuming a low-saturated fat diet, compared to either *APOA2^+/+^* or *APOA2*^+/-^ individuals. A third study recently used 4,602 individuals obtained from 2 additional populations (a high-cardiovascular risk Mediterranean population with 907 men and women, and a multiethnic Asian population with 2,506 Chinese, 605 Malays, and 494 Asian Indians). This study was specifically designed to investigate possible interactions between the *APOA2* SNP (rs5082) and consumption (≥ 22 g per day) of saturated fat [92]. Consistent with the previous two studies, the results indicated a significant interaction between *APOA2^-/-^* individuals and consumption of saturated fats in relation to an increase in BMI in the 2 populations; these individuals had a 6.8% greater increase in BMI when consuming a high-saturated fat diet, but not when consuming a low-saturated fat diet, compared to either *APOA2*^+/+^ or *ApOA2*^+/-^ individuals. Therefore, these results obtained from three independent and diverse populations with large sample sizes provide the first consistent evidence of a gene-diet (*APOA2* gene and saturated fat) interaction in promoting increased weight gain and human obesity, although the molecular basis for the weight gain remains undefined. 

## CONCLUSION

Researchers now appreciate that common obesity represents a complex multi-factorial disease resulting from the interaction of susceptibility genes with an obesogenic environment characterized by increased consumption of energy-dense foods or specific nutrients and a sedentary lifestyle. Given the current epidemic of childhood obesity, continued investigation of gene-diet interactions will be important for several reasons. First, most diseases, if not all diseases, and associated outcomes result from an undefined and complex interaction between susceptibility or modifying genes and various environmental factors [17, 93, 94]. With respect to the susceptibility genes outlined in this review, each gene interacts with specific macronutrients (high-fat food or high saturated-fat food) to promote obesity [95]. Second, the search and identification of gene-diet interactions should be at the forefront in attempts to understand both the etiology and pathophysiology of nutrition-related diseases, particularly childhood obesity [96]. And third, the interaction of specific genetic variations with a known dietary component could eventually lead to more detailed population-based studies to identify and provide targeted treatment for individuals at increased risk of developing childhood obesity [97, 98]. The goal for investigating gene-diet interactions would be to abandon the erroneous notion that for potential nutrition-based interventions “one size fits all.” Instead, a plausible mechanism-based approach to personalized nutritional and/or medicinal therapy will more effectively address the current epidemic of childhood obesity.
